# Cosmology of belonging: The role of community in the therapeutic use of psychedelics

**DOI:** 10.1017/S1478951524001688

**Published:** 2025-01-21

**Authors:** Caroline Dorsen, Lola Noero, Michelle Knapp, Kristin Arden, William E. Rosa

**Affiliations:** 1 Rutgers School of Nursing, Newark, NJ, USA;; 2 Psychotherapist, New York, NY, USA;; 3 Psychiatric Nurse Practitioner, New York, NY, USA;; 4 Lead Clinician, Mindbloom, New York, NY, USA; 5 Assistant Attending Behavioral Scientist, Memorial Sloan Kettering Cancer Center, New York, NY, USA

**Keywords:** Belonging, community, ethnography, group process, group therapy, psychedelics, qualitative

## Abstract

**Background.:**

The recent wave of clinical trials of psychedelic substances among patients with life-limiting illness has largely focused on individual healing. This most often translates to a single patient receiving an intervention with researchers guiding them. As social isolation and lack of connection are major drivers of current mental health crises and group work is expected to be an important aspect of psychedelic assisted psychotherapy, it is essential that we understand the role of community in psychedelic healing.

**Objectives.:**

To explore how psychedelic guides in the United States discuss the role of “community” in naturalistic psychedelic groups.

**Methods.:**

This is a secondary qualitative data study of data from a larger modified ethnographic study of psychedelic plant medicine use in the US. Fifteen facilitators of naturalistic psychedelic groups were recruited via snowball sampling. Content analysis was used to identify themes.

**Results.:**

Participants viewed the concept of community as essential to every aspect of psychedelic work, from the motivation to use psychedelics, to the psychedelic dosing experience and the integration of lessons learned during psychedelic experiences into everyday life. Themes and subthemes were identified. *Theme 1*: The arc of healing through community (*Subthemes*: Community as intention, the group psychedelic journey experience, community and integration); *Theme 2*: Naturally occurring psychedelic communities as group therapy (*Subthemes [as described in* Table 2]: Belonging, authenticity, corrective experience, trust, touch).

**Significance.:**

Results suggest that existing knowledge about therapeutic group processes may be helpful in structuring and optimizing group psychedelic work. More research is needed on how to leverage the benefit of community connection in the therapeutic psychedelic context, including size and composition of groups, selection and dosing of psychedelic substances in group settings, facilitator training, and role of community integration. Psychedelic groups may provide benefits that individual work does not support.

## Introduction

An estimated 17% of adults in the United States (US) have used a psychedelic drug, including psilocybin, Lysergic acid diethylamide (LSD), 3,4-Methylenedioxymethamphetamine (MDMA), or another serotonin agonists, at least once in their lifetime, (Krebs and Johansen 2013) with recent data showing steadily increasing prevalence and frequency (Killion et al. 2021). The potential of psychedelic drugs to address the symptoms of numerous difficult-to-treat mental health issues has garnered substantial scientific interest over the past decade. Broad areas of scientific interest in psychedelic-related outcomes include anxiety, depression, post-traumatic stress disorder (PTSD), obsessive-compulsive disorder (OCD), and addiction (Andersen et al. 2021; Griffiths and Grob 2010; Muttoni et al. 2019).

Patients with serious illness in the palliative care context have proved an especially important population for psychedelic-assisted therapy interventions given their high symptom burden and risk for a compromised quality of life, often compounded by decreased functional independence, autonomy, and agency (Bhagavan et al. 2024; Wang et al. 2024). In an online population-based survey of psilocybin-assisted therapy’s social acceptability for existential distress at the end-of-life among 2800 adult respondents across 4 Canadian provinces, 79.3% of respondents considered psilocybin assisted therapy a reasonable medical choice, 84.8% agreed that costs should be covered by the public health system, and 63.3% would welcome its legalization for medical purposes (Plourde et al. 2024). Common symptoms in the palliative care population may include spiritual and existential distress, anxiety, depression, demoralization, and hopelessness, in addition to a sense of isolation, disconnection, and loss of meaning – complex symptoms and experiences that psychedelic-assisted therapy has been shown to address.

Empirical psychedelic research to date has largely focused on individual treatment in clinical trials, delivering the intervention to a single “client” with therapeutic and research teams guiding the process. This dominant individualist structure exists in stark contrast to collectivist healing modalities of various indigenous psychedelic traditions (e.g., ayahuasca ceremonies, peyote churches) that have historically recognized the power of community as a healing force (George et al. 2020; Metzner 2015). Within the broader realm of society, there is a growing body of literature showing the negative physical and mental health implications of social isolation – particularly following the COVID-19 pandemic, as well as increased emphasis on the role of community in promoting increased psychosocial support and well-being across the life course (Alsarrani et al. 2022; D’Eer et al. 2022; Naito et al. 2023; Liebermann et al., Alsarrani et al. 2022; Prommas et al. 2022; Sallnow et al. 2022). Thus, community connection and group therapy practices that support it may be particularly important to healing for those with serious illness in the post-pandemic era.

Data emphasizing the importance of community connection in psychedelic experiences is growing (Dames et al. 2022; Forstmann et al. 2020; Kettner et al. 2021; Watts et al. 2022). For instance, researchers have reported significant improvements in depression, anxiety, PTSD, and work/life functionality using a community of practice model in a ketamine-assisted intervention for multidisciplinary health workers (Dames et al. 2022). MDMA- and LSD-group therapy over time has suggested group-level development (e.g., mutual support outside of therapy, friendships, conflict, and alterations in individuals’ relationships to others in a population with trauma-related disorders (Oehen and Gasser 2022). Group therapy approaches to psilocybin-assisted therapy in patients with advanced cancer have been shown to be feasible and acceptable to participants (Beaussant et al. 2024). Furthermore, group psilocybin-assisted therapy approaches for patients with cancer and major depressive disorder have also demonstrated safety, feasibility, and efficacy based on clinically meaningful reductions in depressive symptoms (Agrawal et al. 2024). Ayahuasca’s ritual spaces, which emphasize collective consumption, ethics of care, and interpersonal caregiving, are considered fundamental to its efficacy in the treatment of substance addiction (Cosimano 2021; Dupuis 2022; Fairlamb 2016; Goldberg and Hoyt 2015; Joshi 2022; Phelps 2017; Phelps and Henry 2021; Talin and Sanabria 2017).

Outcomes and experiences associated with the naturalistic use of psychedelics (i.e., nonclinical setting use), often communal and relational in nature, have been both undervalued and understudied in the field (Dorsen et al. 2019). However, limited available research suggests that increased social connectedness and self-reported transformative experiences mediate the elevated positive mood associated with psychedelics. Moving forward, the influence of group work before, during and after psychedelic experiences will be the subject of much interest as the scientific community considers how to promote equity, access, and scalability of psychedelic-assisted psychotherapy once Food and Drug Administration (FDA)-approved (National Academies of Sciences, Engineering, and Medicine 2022).

To this end, we conducted a secondary analysis of existing qualitative data to explore the perception of the role of community in “underground” psychedelic groups (Dorsen et al. 2019) in the US. Core therapeutic forces of effective group therapy, based on the formative work done by Yalom and Crouch (1990), were applied to guide data analysis, as described in methods below (see Table 1). Findings describe the role of community and relational health in the context of psychedelics while filling a substantial literature gap. For this study, we use the term “group” to reflect a collection of people who come together to use psychedelics with the stated intention of healing. In some cases, this is once, but in many cases, it is repeatedly over months, years or even decades. We use the term “community” to reflect the interpersonal relationships formed in these groups and the larger group of psychedelic users nationally and internationally.

## Methods

The aim of this secondary qualitative analysis was to explore how facilitators of naturalistic psychedelic groups in the US described the role of community in psychedelic health and healing. This study utilized a modified ethnographic methodology incorporating in-depth, semi-structured interviews with individuals facilitating psychedelic ceremonies in diverse geographic regions of the US. In lieu of traditional participant observational ethnographic methods, the modified ethnographic approach in this study conducted interviews over the phone in order to ensure participant anonymity and facilitate study participation (Dorsen et al. 2019; Rashid et al. 2015). In keeping with ethnographic methods, the study did not incorporate a theoretical framework a priori (Rashid et al. 2015). However, the well-researched and clinically utilized core therapeutic forces of effective group therapy (Yalom and Crouch 1990; Yalom and Leszcz 2020) were used to structure data analysis (See Table 1) and frame the findings in the context of existing literature.

The parent study was approved by New York University Institutional Review Board (IRB) and included a sample of 15 participants who were recruited via key informants and snowball sampling. Inclusion criteria included the following: 18 years of age or older, English speaking, and currently facilitating naturalistic psychedelic group ceremonies within the US. Participants with knowledge of the psychedelic community, culture, protocol, and controversies were selected. As part of a larger study on the growing phenomenon of psychedelic use in the US, the study principal investigator developed an interview guide which addressed topics such as how individuals perceived the purpose and philosophy behind psychedelic ceremonies, the perceived benefits of therapeutic psychedelic use, the perceived risks and limitations of use, similarities and differences between psychedelic-assisted psychotherapy and other modalities of treatment (including individual and group talk therapy), and the differences between recreational and ceremonial drug use (Dorsen et al. 2019).

Interviews were conducted between January and June of 2016 and lasted between 60 and 120 minutes. Following the approved IRB protocols, interviews were recorded and transcribed by a professional transcriptionist. Participants of this study gave verbal informed consent to protect anonymity and demographic data was collected categorically (for example, decade of life rather than age in years). Audio recordings and transcripts were housed on a password-secured computer in a locked office without any identifying information.

Data saturation was met after 15 formal interviews. Directed content analysis was facilitated by use of Dedoose © online qualitative data program to sort and manage qualitative data. Content analysis of the interviews included multiple readings of the transcripts and lengthy discussion among the research team members to illuminate recurring patterns and themes related to community. To this end, transcripts were coded in a multistep process. First, a list of keywords was identified by the research team and transcripts were coded by terms such as community, group, relational, support, and friend/friendship. Next, codes were grouped into categories developed both inductively from the data and deductively from the 11 therapeutic forces in effective group therapy developed by Irvin Yalom, a pioneer of group therapy (See Table 1) (Yalom and Crouch 1990). Finally, categories were condensed and organized into 2 thematic topics with multiple subthemes. Throughout the analysis process 25% of transcripts were coded by 2 researchers to establish inter-coder reliability and ensure qualitative rigor (Squires and Dorsen 2018). To further ensure rigor, direct exemplar quotations from participant’s interviews are highlighted throughout this paper to present participant’s opinions and experiences in their own words. Occasionally quotations have been edited to remove verbal pauses without altering the quotation’s meaning or context.

## Results

### Study participants

The sample in this study consisted of 15 psychedelic group facilitators who collectively have worked with thousands of participants using various psychedelics, including ayahuasca, psilocybin, San Pedro and MDMA. The sample was 80% white (*n =* 12) and 66.6% female (*n =* 10) with 100% of participants having a Bachelor’s degree or higher. Participants had diverse religious backgrounds and professional identities, lived throughout the US and had been leading psychedelic groups for a minimum of 3, and a maximum of 30, years. Of importance, many participants in this study both lead psychedelic sessions and have participated in psychedelic sessions themselves. Thus, their stories sometimes related to others’ experiences and sometimes to their own.

Qualitative findings are categorized into themes and subthemes as follows – *Theme 1*: The arc of healing through community (*Subthemes*: Community as intention, the group psychedelic journey experience, Community and integration); *Theme 2*: Naturally occurring psychedelic communities as group therapy (*Subthemes [as described in* Table 2]: Belonging, authenticity, corrective experience, trust, touch).

### Theme 1: The arc of healing through community

The role and importance of community was reflected in all aspects of participants’ stories in this study and mirrored the arc of the journey^1^ experience from intention setting to psychedelic participation to ongoing integration after their psychedelic experience. Numerous participants in this study considered community to be one of the main efficacy pathways of psychedelics. As one participant stated

For me, [psychedelics are] about remembering and re-experiencing safe connections with other humans … There is so much disconnection in the world … so when I meet new people and I’m describing just the beginning of what this offers, I personally emphasize that the healing, the unique healing that comes from doing this work, is community.

Another participant echoed this perspective, reflecting that the individual and societal need for psychedelic work comes from lack of connection, and the potential power of psychedelics lies in their ability to help heal a disconnected world:

I think that [community] is something we’re missing as a modern society, and we need that again. We’re so driven away from our families and … where we came from … we need to feel connected again. And I feel like [psychedelics are] a tool that could help with that.

### Community as intention

Intention setting is a key aspect in the therapeutic use of psychedelics (Dorsen et al. 2019) and refers to participants’ motivations for participation in therapeutic psychedelic journeys. Intentions may be narrowly focused on what a participant hopes to get out of a specific psychedelic experience and/or address the bigger picture of what a participant is seeking to clarify or change in their lives. Intention setting may lend a structure to what is experienced in psychedelic journeys and/or help frame a narrative for interpretation of the experience in the days, weeks, or even years afterward (Frecska et al. 2016). In this study, participants often stated that community figured into their own motivation for working with psychedelics and/or was a common intention for those they were guiding in psychedelic groups. One participant described this by saying that they personally chose to take psychedelics because they “were looking for a place of tribe.” Another study participant explained that they had been “looking for [community] for all [their] life” and that they found it in a psychedelic community.

### The group psychedelic journey experience

Participants in this study also spoke about the role of community and connection during the psychedelic experience itself. Some study participants described the “safe space” of psychedelic communities as a driver of positive experiences during journeys. One described a journey as “supportive and nurturing … relational space[s]” where there is “reciprocity and mutual respect.” Another participant explained that during a journey “you experience this feeling of belonging, you feel connection, you feel enchanted with everybody that’s there” and that this experience creates an opportunity for relearning how to trust and connect on a deeper, more meaningful level. However, others spoke about connection and community in journey space as being most powerful during difficult experiences that commonly occur when taking psychedelics, such as reliving trauma or experiencing fear. In one example, a facilitator spoke about how community helps participants make meaning of these difficult experiences, thereby allowing them to reframe the experience as important and powerful (as opposed to harmful or traumatizing). They stated the following:

[You may fall apart during a journey] but you fall apart in community, you’ve got people holding you, you’ve got people asking you questions … allowing [trauma] to flow so that it can move through you and out the other side, and you can come out shiny and clean from the experience.

Another study participant described being there for others during difficult experiences as part of the way that psychedelics may also change painful narratives for those who serve in the role of supporter:

it’s really about being a compassionate witness, I am here to support you in anything you want to say or do, and being love and safety for them.

### Community and integration

Finally, participants in this study described the role that community plays in psychedelic integration. Integration is an essential part of the therapeutic use of psychedelics and refers to the ongoing “process in which the patient integrates the insights of their experience into their life” (Gorman et al. 2021). In this study, participants commonly reflected on the healing experience of being in a supportive community as one of the lasting benefits of working with psychedelics. One participant described participating in a psychedelic circle as a

corrective experience that … creates a template that I can take into my own life.

Another stated

the healing comes specifically through the community portal, through recreating a sense of tribe and connection between people that you can trust and that you know is going to continue. And that to me is one of the unique things that this community offers.

### Theme 2: Naturally occurring psychedelic communities as group therapy

Along with describing the role of community occurring throughout the arc of the psychedelic experience, participants in this study spoke about the specific multidimensional ways that group psychedelic experiences aided in their healing process. The majority of these categories corresponded directly to therapeutic concepts well known in traditional talk-based group therapy and included belonging, authenticity, corrective experience, and trust (see Table 2).

One participant described psychedelic groups as “a cosmology of belonging” illustrating the multifaceted ways that therapeutic groups function to ensure that all feel welcome, seen, heard, and respected while also allowing for at-times supportive and at other times corrective individual interpersonal learning. Some participants in this study, who themselves work as traditional psychotherapists when not guiding psychedelic ceremonies, commented on the similarities and differences between psychedelic group work and traditional therapeutic work. When describing why they felt psychedelics offered something different than traditional talk therapy, one participant stated

the huge component in this work has been around community. And as a psychotherapist, I felt like wow, there really are limitations around all of the boundaries and the lack of community [in traditional talk therapy]. And I understand that those things are in place for good reason – I implement them with my clients on a daily basis. However, because my trauma happened in the context of relationship, there’s certain things that I feel come up in the context of the community where I can get feedback and I can draw on the resources of community in a way that I’ve never really been able to [in talk therapy].

However, in contrast to most traditional group talk-based therapeutic processes that do not involve the use of touch, some participants in this study spoke about the role of nonsexual touch in psychedelic group work. One participant described the intentional use of touch to create a physical sense of safety to optimize the therapeutic process, stating

I work with people and hold them in love and safety so that they can go within themselves and do what it is they need to do.

Another described the role of touch within the group as

equally important as any kind of intellectual understanding we have. The physicality and the desire for our mammalian bodies to be up against each other’s bodies, is so outstanding in the journey space, it’s like how come in my everyday life, I can’t, I don’t access that?

## Discussion

This secondary qualitative data study was built on a larger modified ethnographic study of psychedelic use in the US. The original study concluded that participants in that small, qualitative study saw psychedelic use as “a self-care and healing modality that is used in the context of community and ritual” (Dorsen et al. 2019). The present study sought to explore how participants discussed the role of community in this context, considering: (1) that social isolation and lack of connection are major drivers of the current mental health crisis in the US and globally, (2) that the majority of promising clinical trials on psychedelics for these mental health problems have been individually focused, and (3) that group work may be an important aspect of psychedelic-assisted psychotherapy as we work to ensure equitable access to these treatments should they be FDA-approved. These themes are particularly important for people with serious illness in the palliative care context where equitable access to mental and existential health interventions, such as psychedelic-assisted therapy, are lacking.

Participants in this study saw community as an essential aspect of every aspect of naturalistic psychedelic work, from the motivation to use psychedelics, to the psychedelic dosing experience itself, to the integration of lessons learned during psychedelic experiences into everyday life. Much of this has not previously been fully described in the literature. A 2019 historical review (Trope et al. 2019) found that group settings were commonly used in the early days of Western psychedelic research, and may have improved participant satisfaction, patient outcomes and cost effectiveness but identified major gaps in the literature. A more recent web-based quantitative study by Kettner et al. (2021) used an adaptation of the *Communitas Scale* to examine the correlation between a sense of “togetherness and shared humanity” during ceremonies and long-term mental health outcomes, finding that community connection during psychedelic ceremonies was positively correlated with participant well-being after ceremonies.

There is a larger body of literature describing the role of community, both generally and in the context of psychedelics, in the indigenous communities who have been using these substances for millennia in rituals, ceremonies, and rites of passage (Stein et al. 2022). Among many indigenous communities “ … human lives are interdependent with and contingent on living in ethical relations with other people, with our ancestors, with plants and animals, and with the natural world overall. Indigenous systems of relationality are the heartbeat of Indigenous existence. They help to illuminate approaches to physical, intellectual, emotional, and spiritual health” (Elliott-Groves et al. 2020). Recognizing and honoring indigenous ways of knowing may help frame the structure and experience of clinical psychedelic work, while reminding participants, healthcare workers and researchers of the need for humility and grace in this work.

Participants in this study also articulated their belief that community is an important mechanism for how psychedelics work to promote health and healing via decreased isolation and increased reciprocal and authentic connection, trust and care. This is an important finding as clinical trials have almost exclusively been individually based and hypotheses regarding pathways of efficacy of psychedelics have primarily focused on the spiritual (mystical experiences) (Johnson et al. 2019) structural neurobiological (neural plasticity) (Calder and Hasler 2023) and biochemical (serotoninergic) (Carhart-Harris 2018) rather than the relational. The findings of this study suggest that relational health may be an important pathway to explore moving forward and that administering psychedelics in community may not be a “good but lesser” option (as compared to individual work), but rather may offer benefits that individual work does not and/or be more efficacious than individually situated care (Kettner et al. 2021; Liebmann et al., 2022). More research is needed on how to optimize group use of psychedelics, including size and composition of groups, selection and dosing of psychedelic substances in a group context, training of facilitators and ongoing integration (National Academies of Sciences, Engineering, and Medicine 2022). Of note, studies among patients with serious illness are particularly important to not only meet a pressing need for symptom and distress management, but also optimize intervention delivery that decreases system, therapist, and administrative burden while providing pragmatic and convenient opportunities for therapeutic (Agrawal et al. 2024; Beaussant et al. 2024).

Community as medicine in other aspects of health and healing, such as in 12 step and other groups formed to support people with substance use disorder, has been well described in extant literature (Kelly et al. 2020). For example, group talk therapy has long been a leading treatment modality for mental health concerns including: major depressive disorder, bipolar disorder, panic disorder, PTSD, social phobia, OCD, bulimia nervosa, binge-eating disorder, substance use disorder, schizophrenia, borderline personality disorder, and general personality disorders (Lucre and Corten 2013) (Brennan et al. 2014) (Feng et al. 2012) (Wolgensinger 2022). Group therapy explores an individual’s social tendencies and allows for healing within a supportive, socially connected context (Whittingham 2018). Among populations with serious illness, approaches such as meaning-centered group psychotherapy (MCGP) have shown increased sense of meaning and spiritual well-being, as well as decreased anxiety, depression, hopelessness, physical symptom distress, and desire for hastened death (Breitbart et al. 2015). Although researchers have found that some short-term effects of MCGP on participants faded with time (e.g., personal meaning, some effects related to psychological well-being), the long-term positive effect on positive relations with others was sustained (Holtmaat et al. 2020). Thus, as psychedelic-assisted therapy interventions are increasingly implemented in palliative and supportive care contexts, it is essential to assess the feasibility and appropriateness of group therapy settings, and combined efficacious interventions such as MCGP in addition to psychedelic-assisted therapy, to enhance the possibility of lasting benefit and a sense of connection with others, which may bolster personal growth and coping resources.

Findings in this study suggest that existing knowledge about therapeutic group processes used in these other contexts may be helpful in structuring and optimizing group psychedelic work moving forward (Agrawal et al. 2024; Beaussant et al. 2024; Wang et al. 2024). Central themes in this study, including belonging, authenticity, corrective experience and trust, correspond to well-documented concepts of effective therapeutic group therapy (Yalom and Crouch 1990) (Agrawal et al. 2024; Beaussant et al. 2024; Wang et al. 2024). These include universality, group cohesiveness, instillation of hope, interpersonal learning, corrective emotional experiences, and development of socialization techniques (See Table 2) (Yalom and Crouch 1990). Implications for palliative care and psychedelic-assisted therapy research can be found in Table 3.

However, as researchers and clinicians consider the potential benefits of group work in the context of psychedelics and palliative care, it is essential to also that they also consider the potential pitfalls and negative outcomes of group work previously described in the literature. These include decreased confidentiality, decreased accountability, less flexibility, decreased therapeutic alliance, less focused individual attention, a poor fit between a participant and therapist or other group members, and the ability for individuals to “hide” in groups (Yalom and Leszcz 2020). Future qualitative and quantitative research should measure both positive and negative outcomes of group work and consider interventions to optimize the therapeutic potential of groups while minimizing potential pitfalls.

## Limitations

Limitations related to design, analysis, and sample are essential to consider. First, the study was a secondary analysis of existing data, (Dorsen et al. 2019) preventing follow-up or clarification of content with interview participants. For example, we could not ask more detailed questions about psychedelic participation, such as how often participants attended groups and/or if they had also participated in individual sessions. As well, the potential harms or risks associated with group work in this context were not explored in the parent study but will be important to explore moving forward. In this study, consistent data was identified, allowing for therapeutic saturation within 15 interviews. However, the snowball sampling method used in the original study could have resulted in sampling bias, leading to premature data saturation. The results of this small qualitative study are not meant to be generalizable but rather offer a window into a complex phenomenon and community. Next, we used the core components of the group therapy dynamics to guide data analysis, which ensured that findings from this study could be considered in the context of what is already known about group therapy generally. However, this decision may have prevented a more inductive interpretation of available thematic content. While this analytic approach may have potentially led to the exclusion of important experiences, the findings contribute to filling the notable gap related to well-established group therapy forces and the role of community in psychedelic-assisted therapy. Finally, participants predominantly identified as white and consisted of psychedelic group facilitators, limiting diversity in perspective and cross-cultural insight.

## Conclusion

Through shared values and experience, and the changing relationships to self, other, nature, and spirit often seen in psychedelic use, psychedelic communities may optimize the healing power of psychedelics while also offering a structure to improve access for all. Future research should focus on how to minimize any potential harms and best optimize community experiences for the benefit of individuals, families, and society at large, particular among those with serious illness. Community connection in the psychedelic-assisted therapy milieu can become yet another therapeutic tool to garner social support, empathy, and understanding while alleviating the sense of isolation and despair that often accompanies the serious illness experience.

## Figures and Tables

**Table 1.** Yalom therapeutic forces in group therapy (Yalom and Leszcz 2020)

**Instillation of hope**: encouragement that recovery is possible by sharing stories and information.
**Universality**: recognition of a shared experience and knowing a person’s problems are not unique.
**Imparting of information**: teaching about problems and learning factual information about treatment options.
**Altruism**: helping and supporting others by experiencing the ability to help another person can build self-esteem. It helps to develop adaptive coping mechanisms.
**Simulation of the primary family**: identifying & changing the dysfunctional patterns or roles one played in the primary family.
**Development of social skills**: learning new ways to talk about feelings, observations, and concerns.
**Imitative behavior**: modeling another’s manners & recovery skills.
**Interpersonal learning (modeling, vicarious learning**): finding out about themselves & others from the group. Yalom also describes 3 important concepts with interpersonal learning; (1). the importance of interpersonal relationships, (2). the corrective emotional experience, and (3). the group is a social microcosm.
**Group cohesiveness (belonging**): the feeling of belonging to the group, and valuing the group.
**Catharsis**: the release of emotional tension i.e. a burst of crying. Express emotions in a safe environment.
**Existential factors (risk, responsibility):** Learning to take responsibility for one’s own actions.

**Table 2.** Subthemes, exemplar quotes, and corresponding group talk therapy concepts

Subtheme	Exemplar quote(s)	Group therapy concept(s)
**Belonging**	“This work is the cosmology of belonging” “You experience this feeling of belonging, you feel connection, you feel enchanted with everybody that’s there. You want to touch them, you want to bond with them, you want to share in their enjoyment of the same moments. And for a lot of people, that is not a normal feeling for them.”	Group cohesiveness Interpersonal learning Universality
**Authenticity**	“Through the experience of being able to be yourself on (psychedelic) medicine, it allows you to connect with people in a way to where you’re not questioning whether they really get you” “I believe the purpose of the work is to find your authentic self … so that you are actually a part of the community and there’s a synergistic balance … I think this work allows you to be authentic and heal at the cellular level.”	Instillation of hope Interpersonal learning Universality
**Corrective experience**	“Coming from trauma, where there was no safe container … it’s almost like [this is] a corrective experience, in that there is a container, and I can express this stuff, and it feels supportive and nurturing in a way that … it almost creates a template … that I can then take into my own life.” “For me, [psychedelic groups are] about remembering and re-experiencing safe connections with other humans … recreating a sense of tribe and connection between people that you can trust and know is going to continue. And that to me is one of the unique things this community offers.” “Because my trauma happened in the context of relationship, there’s certain things that, you know, I feel that come up in the context of the community where I can get feedback and I can draw on the resources of community in a way that I’ve never really been able to before.”	Corrective emotional experience Universality
**Trust**	[Journeys] “create a safe space to individually and as a community, to really investigate who you are, who we are as people, what our community is. It just creates a safe space.” “It’s really about being a compassionate witness, I am here to support you in anything you want to say or do, and being love and safety for them.” [Journeys} “feel supportive and nurturing … relational space where there are other people who feel like emotionally present … a reciprocity and mutual respect.” “One of the most important pieces [is] to help people feel safe being vulnerable, which doesn’t mean putting themselves into unsafe environments … but being willing to open up emotionally to people and the physical world, nature, structures …”	Develop socialization techniques Instillation of hope Interpersonal learning
**Touch**	“When I felt that energy pouring into me (in a psychedelic group), I was holding a woman who was actually older than I was, and I didn’t say anything out loud. And all of a sudden, she said “oh my god, is this what a mother’s love feels like?” “And this is much more of a social event, and it’s giving us an opportunity to be in the spirit of plant medicine in a social context where we can talk about things … and are in each other’s physical presence in ways that we’re not that way in the regular world, it’s a lot more touching. And that piece is equally important as any kind of intellectual understanding we have. The physicality and the desire for our mammalian bodies to be up against each other’s bodies, is so outstanding in the journey space, it’s like how come in my everyday life, I can’t, I don’t access that?” “I work with people and hold them in love and safety so that they can go within themselves and do what it is they need to do.”	(Not applicable)

**Table 3.** Implications for future palliative care and psychedelic-assisted therapy research

Naturalistic psychedelic facilitators and guides, as well as inter-professional therapists and teams, should elicit the meaning and importance of community to the client to best cocreate a supportive and person-centered set and setting. Additional research is needed with a broad range of individuals and groups to explore the meaning and role of community. For example, the concept of belonging is subjective and defined in individual terms based on worldview and history. Collectivist cultures may value community in nuanced and concrete ways that contradict the staunch individualism often normalized in Western cultures. Approaches that prioritize community engagement and priorities in research, such as community-based participatory research, will be needed to ensure communities as equal partners in all aspects of the research process while ensuring community-specific values, needs, and goals drive scientific design, implementation, and dissemination. Whole-family and whole-community research approaches should be actively encouraged. For instance, there is increasing global attention on the role of compassionate communities to de-medicalize death, dying, and grief while increasing social cohesion, pragmatic support, cultural understanding, and accompaniment for patients and families experiencing life-limiting illness or injury. (Sallnow et al. 2022) These communities may provide crucial support structures to buoy sustained, community-based psychedelic integration for patients post-psychedelic dosing. Numerous participants in this study spoke about the role of nonsexual touch as vital and beneficial. Touch has been seen as a problematic practice in psychedelics given the extreme vulnerability of people with altered consciousness. (Rouhe 2022; Villeneuve and Prescott 2022) Client accusations of therapists’ immoral, harmful, and inappropriate sexual touch in recent years have led to myriad questions about client-therapist boundaries and physical closeness. Yet, experts consistently agree that touch can be useful, comforting, grounding, and reassuring if consent and practice is discussed and agreed upon between client and therapist during preparation phases and prior to psychedelic administration. Research is needed to explore if touch can be safely incorporated into psychedelic work, and, if so, how informed consent and ethical standards will figure into its use. (Gearin et al. 2021)

## Footnotes

1 Journey is the word commonly used for psychedelic trips or experiences.
